# Supplementary material for: The psychological impact of torture and state repression in Türkiye between 2015 and 2018: Reports from Turkish refugees seeking asylum in Germany
Source: PLOS Glob Public Health. 2024 Jul 29;4(7):e0002561. doi: 10.1371/journal.pgph.0002561 (PMC11285925; doi:10.1371/journal.pgph.0002561)
Supplement: S1 File — (DOCX) [file pgph.0002561.s001.docx]

**S1 File: Quotes in the original language Turkish in order of appearance**

*Torture*

*Dedim ya işkencenin yöntemlerinden bir tanesi de İstiklal Marşı okunuyor mesela (...). Ama orada bir çalıyor, bir daha çalıyor, bir daha çalıyor, bir marş çalıyor, ardından Mehter Marşı derken artık böyle çıldıracak hale geliyorsunuz. Birde o yüksek ses, sizi delirtmek için uğraşıyorlar, beyin yıkaması yapıyorlar. (TN21, Pos. 35)*

*93 gün boyunca yaşadıklarım ve duyduklarım, bana sorulan sorular, soru soran kişilerin tavırları, mesleki deneyimleri, teknolojik olarak ulaşabildikleri olanaklara bakınca bunlar bir çete, üç – beş tane serseri değil, bir organize, nöbet değişimleri olan, bilimsel olan işkence metodlarının her birinin uygulandığı bir işkence merkezine götürüldüm. Ben hani “belki öldürülürüm”... kafamda zaten... (TN21, Pos. 36)*

*Arbitrariness and state violence*

*Üniversitenin içinde polis gelip, biz o süreçte ufak tipte politik işlerle uğraşıyoruz... Beni tehdit ediyorlar “baban ve kardeşin içerde, seni de alırız” diye... (TN 12, Pos. 19)*

*Orada da dövdüler, korkuttular ama ikincisi kadar değildi, ikincisinde yani Ekim’de o zaman Rojova’ya saldırı vardı, Türkiye’nin Rojova saldırısı, dedi “biz seni sınıra götürürüz, kafana da sıkarız, teröristtir diye öldürdük” deriz. Şimdi yaparız bunu dediler. (TN 16, Pos. 61)*

*Police house searches*

*Önce yan odadaki arkadaşlarımıza yapılan işkenceler sonucu onların çığlıklarına maruz bırakıldık. “Birazdan size geleceğiz, sizin üzerinizde ter atacağız, sonra size de aynı çığlıkları attıracağız” gibi psikolojik işkence yapıldı. Zaten kaba dayak, yumruklar, tekmelerin, saç çekmelerin dışında bunları söyleyebilirim. Daha sonra o yan odadaki arkadaşlarımızın kan revan içindeki hallerini gösterip, “buna iyi bakın, bir daha göremeyeceksiniz” diyerek, bizi onun öldürüleceği korkusuna sürüklemek gibi psikolojik işkenceler. (TN 2, Pos. 45)*

*İstanbul’daki olayda mesela darbeden sonra evimiz defalarca basıldı, sabaha karşı kapılarımız kırıldı, içerde yani çok keyfi muameleler, her tarafı dağıtmalar, okuduğum kitaplar resmi basılmış kitap, yasak olmayan ama onlara göre yasak bir kitap. (TN 5, Pos. 5)*

*Annemi sırayla geçmişler, küfür etmişler. Annem hala küfürleri söylemiyor. Sadece “senin için bana çok kötü şeyler anlattılar” diyor. Ne anlattıklarını bilmiyorum. Sonra sıraya geçip annemin yüzüne tükürüyorlar. (TN 10, Pos. 93-94)*

*Violence at demonstrations*

*Zaten orada yapılan bombalamayı devlet terörü olarak adlandırıyoruz. Onun dışında psikolojik olarak ta insanlara işkence yapıldığını düşünüyorum. Çünkü hala, insanlar psikolojik olarak hala kendilerine gelemiyorsalar, intihar ediyorsalar her iki işkencenin de var olması demektir. (TN 17, Pos. 75-76)*

*Tehlike hep vardı, yakalanma... Mesela şu var... Şu an Türkiye’de olsaydım, yakalanırdım, içeri atılırdım ama hiçbir yere bilgi sunulmazdı. Yani polis derdi ki; “bu adam bunu yapmış, şunu yapmış”. Dolayısıyla herkeste böyle bir güvensizlik, böyle bir psikolojik baskı var zaten. Şu anda bu uygulanıyor. Adam istediğini içeri atabiliyor, istediğini öldürebiliyor, ama dediğim bu ağır şeyler de var. Ben bu hikâyelerle büyüdüm. Bu hikayelerden binlercesini dinleyebilirsiniz... (TN 15, Pos. 26)*

*Detention and police custody, charges and prison conditions*

Parmak izi vermek istemiyoruz arkadaşlarla, tartışıyoruz... “İsterseniz verirsiniz, vermezseniz parmaklarınızı kırarak alırız” dediler… (TN 1, Pos. 31-34)

Keza Adli Tıp’a giriyorsun, ordaki doktor da bunun içine katılıyor, fiziksel olmasa da. Yani her yerinde morluklar var, burnundan kan geliyor, ne bileyim kiminin gözü kapanmış açılmıyor filan ama çok net bir şekilde seni hiç muayene etmeden sağlam raporu verebiliyor. (TN 7, Pos. 23)

*Violence in the context of conflicts in Kurdish areas*

Çünkü yaşanan şeyler, normal şeyler değildi. Cesetler bir odunu yakar gibi hepsi kül olmuş. Tanınmıyordu şimdi cesetler, bazıları aynı şekilde hala cesetlerini teşhis edemediler. İşkence, tamam savaştır, savaş dağda, şehirde değildir. Dağdakini de öldürürsün ama o kadar işkence, o kadar yakma; insani olarak yani bilmiyorum hiç bir şeye sığdıramıyorsun. (TN 17, Pos. 50)

*Violence of civil government supporters*

Facebook’tan mesaj geldi bana, “Kürt ırkını yer yüzünden sileceğiz” diye. Hem de bireysel olarak tanımadığım bir erkek cinsel organını göndermiş, artık resim olarak atmış ve “Ne mutlu Türküm diyene kadar seni altımda inleteceğim” tarzında bir mesajdı. (TN 13, Pos. 36)

[…], dışarda da fiziksel ya da psikolojik olarak yapılan baskının işkence olduğuna inanıyorum. Bu yüzden aralarında kesinlikle bir bağlantı var. Çünkü algı ve zihniyet bu şekilde. Hani “sizden olmayana istediğinizi yapabilirsiniz, istediğinizi söyleyebilirsiniz” algısı var ve güçlü olduklarına inanıyorlar. Tabii ki arkalarında bir devlet olduğu için gerçekten güçlüler de. Bu yüzden bu da işkence benim için. (TN 13, Pos. 51)

*Sexualised violence*

Her gözaltında tacize, çıplaksın zaten. Yapabileceğin hiçbirşey yok, çıplaksın. İç çamaşırıylasın... (TN 10, Pos. 60)

Hani tecavüz edecekler diye korktum. Beni bir yerde yakalayacaklar, birşey yapacaklar bana... Çünkü Türkiye bu anlamda çok müsait. Yanlız yaşıyorsun, yanlız başına bir kadınsın, ifşa edilmişsin çok tehlikeli bir gazete tarafından, taraftarlarının ne kadar tehlikeli olduklarını biliyoruz. (TN 13, Pos. 36)

Çıplak arama dayatması oldu... Onu yapamadılar ama... Ama bunun tehdit olduğu, aralarda sürekli hakaret ediyorlar mesela... Benim cinselliğim üzerinden mesela... “İstersen kadın polis gelsin” filan iğrenç “espiriler” filan... (TN 1, Pos. 35)

*Acute response*

O An; yani beni artık burada öldürsünler de kurtulayım dedim... Psikolojik olarak; “herhalde daha buradan çıkmam dedim”... Birde “daha 14 gün burada yanımızdasın” diye baskı yapıyor... “Buradan daha çıkmayacaksın”... Yani “artık öldürseler de kurtulsam” diye düşünüyorsun...

Ama belli bir süreden sonra korku kalmıyor artık... Korkunun yerine cesaret geliyor... Galiba böyle düşünüyorsun... “Ben kendim önlem almalıyım... Artık bundan başka daha ne yapabilir ki... En fazla öldürür zaten... Yapacağını yapıyor, başka ne kaldı ki?” (TN 4, Pos. 95-96)

Aslında o esnada düşünmüyorsun. O esnada sadece korkuyorsun. Korku var evet... Korkmuyorum diye cesaret yapmaya gerek yok. Herşeyin bittiğini düşünüyorsun. (…) Yani o anda korkuyorsun, o anda hiçbirşey düşünmüyorsun, hissetmiyorsun, bir an belki boşlukta kalınca belki biraz düşünebilirsin, çünkü senin zaten düşünebilecek bir fırsatın yok ki... Ne düşüneceksin ki? (TN 10, Pos. 110-111)

*Emotional reaction*

Artık herşey çok anlamsızdı bana. Ağlamıyordım da, gülemiyordum da, öyle ara bir ton vardı duyguda. Yani uç bir öfkem vardı, bir yerden sonra o öfke kabuk bağladı. Artık öyle çok can yakan bir öfke değildi,başka birşeydi. Artık herşey anlamsızdı. Ben diyordum “bu saatten sonra Türkiye’nin tümü özgürleşde de, bu despotluk yıkılsa da artık bir anlamı yok” diye düşünmeye başladım. Çünkü çok korkunç şeyler olmuştu. Savaş suçları olmuştu. (TN 15, Pos. 22)

Onlara karşı kendimi suçlu hissediyorum. Evet coğrafyamızda bu stresi, bu sıkıntıları herkes yaşayabilir ama ben birşey için mücadele ettim, inandığım değerler için. Ama diyorum ki, “keşke onları dahil etmeseydim”. Onların dahil olmamasının bir yolu varmıdır diyorum. (TN 16, Pos. 165)

*Fear and anxiety*

Translated version, originally in German: Ve hepimiz şeyden korkuyorduk... Yani sadece iki tane intihar saldırısı olduğunu bilmiyorduk ki. O gün öleceğime emindim. Bundan emindim. (TN 11, Pos. 26)

(…) tedirginlik hep vardı bende açıkçası. Her an birşey olacak, bir yerden çıkacak diye sürekli sağıma soluma bakmak zorunda kaldığım bir dönem oldu maalesef. (TN 13, Pos. 43)

*Anger and Impulsivity*

Bir dönem çok sinirliydim, sonra o sinirlilik duygusallığa dönüştü, sonra bir dönem hiçbirşey hissetmez duruma geldim, hem de herkese ve herşeye karş,ı anne baba da dahil olmak üzere. Şu an ise, o süreçlerden mi kalmadır bilmiyorum ama mesela çok çabuk sinirlenen, hemen öfke patlaması yaşayan ama aşırı derecede de duygusallaşan bir insanım. (TN 6, Pos. 101)

Yani uç bir öfkem vardı, bir yerden sonra o öfke kabuk bağladı. Artık öyle çok can yakan bir öfke değildi,başka birşeydi. Artık herşey anlamsızdı. (TN 15, Pos. 22)

*Powerlessness and Hopelessness*

Yani bekledik, baktık yerde birkaç arkadaş var, bazıları yaralandı bazıları ölmüş o iki tane arkadaş... Biz daha şey yapmadık açıkçası... Yani ben tepki veremedim... Çünkü ne olduğunu tam anlayamadım sadece... (TN 3, Pos. 97)

Translated version, originally in German: Ben o zamanı, yani Ağustos’tan sonraki dönemi, Sur ve Diyarbakır’da, yani Amed’de bir çok insanın öldürüldüğünü hatırlıyorum. Ben;her gece, abartmıyorum, her gece ağladığımı hatırlıyorum. Hep yatağımda. Ağlıyordum, ağlıyordum, ağlıyordum, kendimi güçsüz hissediyordum. Düşündüm, böyle olmaz dedim, bu şartlar altında devam edemem dedim. (TN 11, Pos. 24)

Şu an insanların genelde hepsi iyi niyetli, Avrupa’daki toplum çoğu da bizi anlıyor. İnsanların hepsi kötülüğü düşünmezler ama devlet farklı bir organizmadır ve Avrupa’daki tüm devletler bu olup bitenleri çok iyi bilmelerine rağmen sessiz kalıyorlar. Türkiye ile ticaretlerini sürdürüyorlar, silah satmaya devam ediyorlar, Erdoğan’ı maddi olarak desteklemeye devam ediyorlar. Çünkü Avrupalılar çok analitik düşünüyorlar. Devletler. Yani Türkiye ciddi bir kaos yaşarsa mülteciler daha farklı gelmeye başlar. Türkiye şu an NATO’da dır. Oradan kopup Rusya’ya yaslanırsa Avrupa ve Amerika’nın hesapları karışır. Yani analitik böyle. (TN 15, Pos. 24)

Courage and strength

Birde o yüksek ses, sizi delirtmek için uğraşıyorlar, beyin yıkaması yapıyorlar. Ben de kendime göre o esnada, psikolojik direnişlerden bir tanesi olarak, kendimi meşgul edebileceğim başka şeylere zihnimi yönlendirerek, o gürültünün içinde kendime küçük bir dünya kafamda, zihnimde oluşturup hani direnç oluşturuyordum özellikle. (TN 21, Pos. 35)

Ama belli bir süreden sonra korku kalmıyor artık... Korkunun yerine cesaret geliyor... Galiba böyle düşünüyorsun... “Ben kendim önlem almalıyım... Artık bundan başka daha ne yapabilir ki... En fazla öldürür zaten... Yapacağını yapıyor, başka ne kaldı ki?” Öyle düşünüyorsun artık... (TN 4, Pos. 96)

Düştükten sonra hala yaşadığımı görünce beni camdan atmadıklarını anladım. İnsan bu kadar yaşadıkları şeylere rağmen yaşamak istiyormu, istiyor. Ben istedim. (TN 10, Pos. 62)

Şöyle düşün, en uzak köşelere birbirimize sesimiz de gitsin ki yaşananları bir nebze de ne kadar dışarıya ya da farklı yerlere ulaştırabilirsek yapılan saldırının biraz da vahametini de ortadan kaldırıyor. Ondan sonra zaten sloganmı atıyorsun, işkencemi görüyorsun. (TN 14, Pos. 40)

Long-term consequences

Galiba biraz sinirli oldum herhalde... Dalgınlık var... Bir şeye odaklanmakta zorlanıyorum... Birde yorgunluk hissi... (TN 4, Pos. 108)

Çok ani sinirleniyorum. Ani ağlama nöbetleri geçiriyorum. Yani en ufak, normal böyle gülerken ufacık birşey bana geçmişi hatırlatabiliyor, o anda hemen o ortamdan kopup oraya gidebiliyorum. Elimde değil, buna engel olamıyorum.

Beynim bazen duruyor, hiçbirşey düşünemiyorum. Böyle bir boşluktayım, zorluyorum kendimi, zorluyorum, zorluyorum, hiçbirşey düşünemiyorum. O an orda bitiyor. (TN 10, Pos. 166-167)

Olumlu hayaller de kuramıyorum. Mesela diyelim ki buradan çarşıya gideceğim, mutluluk hayali kuramıyorum, diyorum ki “tersi olacak”. Sanki yapmayı istediğim güzel şeylerin hep tersi olacakmış gibi bir kaygı içindeyim. (TN 16, Pos. 166-168)

Hypervigilance and lack of trust

Çok temel bir güvensizlik duygusu bir kere. Mesela önceleri ben perdemi kapatmadan yatardım ama bu olaydan sonra sürekli birisinin beni izlediği hissi oluştu ve hiç rahat olamıyorsun bir kere... İlk zamanlar bayağı tedirgindim ve sürekli sağımı solumu kontrol ediyordum. (TN 1, Pos. 94)

Ani seslere duyarlılık, bir bardağın burdan düşmesi mesela... Telsiz seslerinin huzursuzluğu, gece ani seslerde veya birinin beni uyandırması. Mesela biri beni uyandırdığında çok panik oluyorum. (…) Yani çok panik hali, sürekli bir dikkat halindeyim. (TN 2, Pos. 73)

Kimseye güvenmiyordum açıkçası... Türkiye’yi terk edene kadar... (TN 21, Pos. 45)

Isolation, loneliness and silence

Ben başkalarına da kötü hissettirmeyeyim, yani onlara da kötüyü yaşattırmayayım diye kimseyle konuşmuyorum da... (TN 12, Pos. 91)

Buna bağlı evde de aynı stresi yaşıyordum, çocuklarıma zarar vermemek için onları üzmemek için odaya girip, kapıyı kapatıyordum. Bu durum beni daha çok yalnızlaşmaya götürüyordu. (TN 16, Pos. 154)

Surpressing feelings and forgetting

Bütün bunlardan kaynaklı da hep kendimden kaçmak istiyorum. Kendimden kaçma isteği sürekli doğuyor. Bu sorunlardan, bu problemlerden sürekli uzak durayım. (TN 16, Pos. 170)

Çünkü insan kendi kendini korumaya alıp şey yapıyor. Mesela sanki hiç olmamış gibi, sende ağır travma yaratan şeyleri, sanki hiç yaşanmamış gibi, o artık sana o kadar çok acı veriyor ki, sen onu dondurup dolaba koyuyorsun hiç yaşanmamış gibi. Bir süre sonra da ona inanıyorsun. (TN 20, Pos. 14)

*Political Conviction and Religious Belief*

Yaşadıklarımız her an daha ağırlaşıyordu. Bu tabii ki siyasi fikirlerimizden, yaptığımız işlerden dolayı. Ama bunu sonucunu bilerek yaptığımız için aslında, tabii nokta atışı bana kesin şöyle davranırlar şeklinde değil ama sıkıntının olabileceğinin bilincinde biraz daha hazırlıklı olabiliyoruz ve aynı zamanda sadece kendimin yaşamadığımı ve daha ağırının da yaşandığını gördüğüm, bildiğim için kendim biraz daha başa çıkabiliyorum. Yani daha ağır yaşayanlar var, onlar atlatabildiyseler, sen de atlatabilirsin, ya da hep birlikte atlatırız. (TN 2, Pos. 101)

Aslında onuru kırılan biz değiliz... Bu onursuzluğu bize yaşatanlar aslında onuru kırılması gerekenler... Çocuklarının yüzlerine nasıl bakıyorlar ki? Aslında düşündüğün zaman onuru kırılan onlar… (TN 14, Pos. 75)

Psikolojik olarak bu duyguların bana yardımcı olduğunu düşünüyorum. Şöyle söyliyeyim mesela; bana sorarsanız “pişmanmısın” diye, hiçbirşekilde pişman değilim. Aynı şeyler başıma gelecek olsa bile yaptığım şeyden vazgeçmemem gerektiğini ilkesel olarak, etik olarak, bir insan olarak insanlık için birşey yapacaksam bunu yapmaya devam etmem gerektiğini düşünüyorum. (TN 21, Pos. 51)

Subjective well-being, work and social activities

Bazen ben böyle Almanya’da dolaşırken ormanda, göl kenarında filan kendimi film izliyormuşum gibi hissederim hala... Bu gerçek mi acaba yoksa yine bir rüya mı? Çünkü ben bir sene hergün rüya gördüm. Hergün rüyamda ülkeyi terk ediyorum, başka bir ülkede yeni bir hayata başlıyorduk ailemle. (…) Durum bu olunca şimdi kendimi yine rüyada gibi hissediyorum. (TN 22, Pos. 48)

Düzenli doğa yürüyüşleri yapıyorum, yemek yemeye çalışıyorum. Ama ben ne kadar yaparsam yapayım bu kamp yaşamı bir şeyden sürekli geri düşürüyor ve kalıyorum orada. Ondan dolayı birşey yapmıyorum. . (TN 8, Pos. 178)

Yaşananlar zaten yaşanmıştır. Hiç birşeyi yaşanmamış gibi görmezsin ama derin bir nefes alırsın. Ama şu anda hala yüreğimi birşey sıkıştırıyor. Nefes alıyorsun ama göğsün dolmuyor. Oh be diyemiyorsun. Birşeyler eksik kalıyor sende. (TN 15, Pos. 24)

Plans for the Future

Genel olarak yaşam motivasyonumun çok düştüğünü hissediyorum. Sabaha uyanmak, güne uyanmak, gün heyecanı yaşamak uzun süredir, yani buraya geldiğimden beri yaşadığım birşey değil... (TN 18, Pos. 107)

Ama burada yapacağımız hep ora odaklı olacağı için bilemiyorum. Ya şu hep içimde ukde kalacak aslında Türkiye’ye gidemediğim sürece. Sokakta elimde fotoğraf makinasıyla koşturmak. O hali aslında... İstediğim o olduğu için, yaptığımız o olduğu için, öyle öğrendiğimiz için aslında... Çünkü aynı zamanda işkence görendik ama işkenceyi de kaydedendik te bir yerde. (TN 2, Pos. 141)
