# Supplementary material for: The psychological impact of torture and state repression in Türkiye between 2015 and 2018: Reports from Turkish refugees seeking asylum in Germany
Source: PLOS Glob Public Health. 2024 Jul 29;4(7):e0002561. doi: 10.1371/journal.pgph.0002561 (PMC11285925; doi:10.1371/journal.pgph.0002561)
Supplement: Attachment — Submitted filename: Response to Reviewers_Rev2.pdf [file pgph.0002561.s003.pdf]

HEIDELBERG
UNIVERSITY
HOSPITAL

Department of General Internal Medicine and Psychosomatics | Thibautstraße 4 | 69115 Heidelberg

Center for Psychosocial Medicine

Department of General Internal Medicine and Psychosomatics

apl. Prof. Dr. med. C. Nikendei, Deputy Chief

Thibautstraße 4
69115 Heidelberg
Tel +49 06221/56-38663
Fax +49 06221/56-3550
christoph.nikendei@med.uni-heidelberg.de

May 15, 2024

Jasmin Lilian Diab, Ph.D

Guest Editor at PLOS Global Public Health

The Bradfield Centre
184 Cambridge Science Park
Milton, Cambridge CB4 0GA
United Kingdom

Second revision of our manuscript *"The Psychological Impact of Torture and State Repression in Türkiye between 2015 and 2018: Reports from Turkish Refugees Seeking Asylum in Germany" for publication PLOS Global Public Health*

Dear Editorial Board of PLOS Global Public Health,

Thank you for the further assessment our article '*Responding to Human Trafficking among Refugees: Prevalence and Test Accuracy of a Modified Version of the Adult Human Trafficking Screening Tool'* for publication in PLOS Global Public Health.

We are pleased about the positive response after the first round of revisions. Enclosed, you will find the second revision of our manuscript, in which the journal requirements and the comment by the editor have been considered carefully. All changes made in the manuscript are listed below as quotations and have also been color-coded in the revised version of the manuscript to ensure that the revisions made are easy to follow.

Thank you for your interest in our article and the opportunity to submit a revised version of the manuscript. We hope for a positive vote and are happy to answer any questions you may have.

Sincerely,
Corresponding author

Estella A. Tambini Stollwerck

Estella Alejandra Tambini Stollwerck, M.Sc.
Research Assistant

Prof. Dr. med. Christoph Nikendei, MME
Deputy Chief
Department of General Internal Medicine and Psychosomatics

Journal Requirements

Journal Requirement No.1

Please review your reference list to ensure that it is complete and correct. If you have cited papers that have been retracted, please include the rationale for doing so in the manuscript text, or remove these references and replace them with relevant current references. Any changes to the reference list should be mentioned in the rebuttal letter that accompanies your revised manuscript. If you need to cite a retracted article, indicate the article's retracted status in the References list and also include a citation and full reference for the retraction notice.

We have checked the reference list and included the articles that are now mentioned in the newly integrated paragraph in response to the editor`s comment (see below). Please note, that no cited paper was retracted.

Journal Requirement No.2

Please send a completed 'Competing Interests' statement, including any COIs declared by your co-authors. If you have no competing interests to declare, please state "The authors have declared that no competing interests exist". Otherwise please declare all competing interests beginning with the statement "I have read the journal's policy and the authors of this manuscript have the following competing interests:"

Thank you for this remark related to further journal requirements. We are pleased to provide more details about competing interests and have included the COIs as proposed, p. 27, line 671-672:

Competing Interests

The authors have declared that no competing interests exist.

Editor Comment

What I find missing from this piece is the framing of 'torture' and torture-related practices in Turkiye. How does the state define, control, or avoid these practices? While the arguments are strong, I think a nuanced understanding of torture remains missing, and framing this conversation against the backdrop of the wider political and human rights discourse/environment in Turkiye remains absent. An independent section on this to introduce the analysis is pivotal. Drawing more on the qualitative data collected would also enhance the rigor of the piece and this angle.

We appreciate the editor's comment on the importance of a nuanced understanding of torture and torture-related practices and control mechanisms in Türkiye. We inserted two headlines to improve the structure on the introduction:

p. 3, line 62:
Torture and torture-related practices in Türkiye

p. 5, line 105:
Literature Review and the Istanbul Protocol

It is self-evident that the state opposes claims on human rights violations that are raised against it. We have now included an official response from the Turkish government to the OHCHR Subcommittee on the Prevention of Torture, in which Turkey sets out its mechanisms for the prevention of torture. At the same time, such sources must always be read critically and compared with the observations of independent bodies, which is why we have also added further sources regarding reports by human rights organisations. We hope we have extended the introduction according to your suggestions:

p. 4, line 71-86:
Amnesty International regularly denounces human rights violations and torture practices (Amnesty International, 2018). The Human Rights Foundation of Turkey (TİHV) describes in its 2022 annual report that torture has been used ubiquitously and systematically in Turkey for years, reporting 1201 survivors of torture approached their centres in 2022 (Human Rights Foundation of Turkey, 2023). Türkiye has signed the United Nations' Convention against Torture and the European Convention for the Prevention of Torture and is therefore obliged to adhere to the absolute ban on torture and to create bodies that take preventive action against possible torture practices. In its official responses to reports over the visits undertaken by the United Nations' Subcommittee on Prevention of Torture and Other Cruel, Inhuman or Degrading Treatment or Punishment (SPT) and the Committee against Torture (CAT), the Turkish government stresses the adherence to the international human rights system and the determination to take effective legislative, administrative and judicial measure for prevention, including the implementation of National Preventive Mechanisms (SPT, 2019). The Turkish government emphasises a 'zero tolerance policy against torture' which had been 'continuously and decisively implemented well over a decade, preventing all forms of torture and other cruel, inhuman or degrading treatment or punishment' (SPT, 2019; p. 11).

p. 5, line 99-100:
[Subsequently, the government imposed a state of emergency that lasted for two years] in which fundamental rights were restricted or even abolished altogether.
