# Supplementary material for: The psychological impact of torture and state repression in Türkiye between 2015 and 2018: Reports from Turkish refugees seeking asylum in Germany
Source: PLOS Glob Public Health. 2024 Jul 29;4(7):e0002561. doi: 10.1371/journal.pgph.0002561 (PMC11285925; doi:10.1371/journal.pgph.0002561)
Supplement: Attachment — Submitted filename: Response to Reviewers.docx [file pgph.0002561.s002.docx]

**Catherine Kyobutungi**

**Executive Editor at PLOS Global Public Health**

PLOS

The Bradfield Centre

184 Cambridge Science Park

Milton, Cambridge CB4 0GA

United Kingdom

**Revised version of our manuscript *“The Psychological Impact of Torture and State Repression in Türkiye between 2015 and 2018: Reports from Turkish Refugees Seeking Asylum in Germany” for publication PLOS Global Public Health***

Dear Editorial Board of PLOS Global Public Health, dear reviewers,

Thank you very much for assessing our article ‘*Responding to Human Trafficking among Refugees: Prevalence and Test Accuracy of a Modified Version of the Adult Human Trafficking Screening Tool’* for publication in PLOS Global Public Health.

We considered the comments and remarks of the reviewers to be very helpful. We believe that the manuscript has improved substantially due to the corrections that have been made. Enclosed, you will find the revised version of our manuscript, in which all suggestions have been taken into account. We address the individual comments point by point in this cover letter. All changes made in the manuscript are listed below as quotations and have also been color-coded in the revised version of the manuscript to ensure that the revisions made are easy to follow.

Thank you for your interest in our article and the opportunity to resubmit a revised version of the manuscript. We hope for a positive vote and are happy to answer any questions you may have.

Sincerely,

Corresponding author

Estella A. Tambini Stollwerck

Estella Tambini Stollwerck	Christoph Nikendei

**Estella Alejandra Tambini Stollwerck, M.Sc.	Prof. Dr. med. Christoph Nikendei, MME**

Research Assistant	Deputy Chief

Department of General Internal Medicine and Psychosomatics
**Reviewer #1**

**The author(s) wrote a manuscript on The Psychological Impact of Torture and State Repression in Türkiye. I believe the study addresses a very interesting, timely and rarely studied topic which appeals to the readership of PLOS. I read it with great interest; however, the manuscript needs substantial revision and structural reframing to be considered for publication. There are a few issues need to be addressed before gets accepted for the publication.**

*Dear Reviewer 1, thank you very much for your appreciation of our manuscript and for highlighting aspects that need to be further elaborated or differentiated. We hope that we have succeeded in implementing the changes you requested.*

**Reviewer 1, Comment No. 1**

**The introduction needs to justify and articulate the focus and questions of this study from the relevant scholarship, policy, and practice perspectives using relevant literature as support. You need to develop research questions at the end of the introduction in alignment with the study focus. Also, there is limited citations from 2017-2023 that APA requires the most recent sources. I recommend to the author(s) to add a few the most recent sources in both literature review and discussion section.**

**Here are some most recent sources that might be very helpful:**

[**https://www.amazon.com/Human-Rights-Turkey-Philosophy-Explorations/dp/303057475X**](https://www.amazon.com/Human-Rights-Turkey-Philosophy-Explorations/dp/303057475X)

[**https://www.tandfonline.com/doi/full/10.1080/14782804.2023.2193876**](https://www.tandfonline.com/doi/full/10.1080/14782804.2023.2193876)

[**https://www.ajqr.org/article/human-rights-conflicts-and-dislocation-the-case-of-turkey-in-a-global-spectrum-8266**](https://www.ajqr.org/article/human-rights-conflicts-and-dislocation-the-case-of-turkey-in-a-global-spectrum-8266)

[**https://www.tandfonline.com/doi/full/10.1080/14767724.2021.2017264**](https://www.tandfonline.com/doi/full/10.1080/14767724.2021.2017264)

Thank you for drawing our attention to these very interesting and relevant research articles. We gladly include these sources into our manuscript as we feel they provide further insight into the current human rights situation in Türkiye and added corresponding citations (see page 4 and 5), for example:

*Dehghan, R., & Osella, C. (2022). The psychological impact of sexual torture: A gender-critical study of the perspective of UK-based clinicians and survivors. Transcultural Psychiatry, 59(3), 380–392.* [*https://doi.org/10.1177/13634615221089491*](https://doi.org/10.1177/13634615221089491)

*Iacopino, V., Haar, R. J., Heisler, M., Lin, J., Fincancı, Ş. K., Esdaile, C., Modvig, J., Sveaass, N., Nathanson, V. H., Melzer, N., & Cohen, J. (2022). Istanbul Protocol 2022 empowers health professionals to end torture. The Lancet, 400(10347), 143–145.* [*https://doi.org/10.1016/S0140-6736(22)00948-5*](https://doi.org/10.1016/S0140-6736(22)00948-5)

*Longstreth, G. F., Attix, C., & Kuck, J. (2023). Torture Survivors and Asylum: Legal, Medical, and Psychological Perspectives. The American Journal of Medicine, 136(3), 244–251.* [*https://doi.org/10.1016/j.amjmed.2022.10.014*](https://doi.org/10.1016/j.amjmed.2022.10.014)

*Patel, N. (2019). Psychological torture. Torture Journal, 29(3), Article 3.* [*https://doi.org/10.7146/torture.v29i3.117412*](https://doi.org/10.7146/torture.v29i3.117412)

Additionally, we stated the research questions as following, see p.5, line 110-117:

*‘The aim of this qualitative research was to examine the psychological impact on vulnerable groups of Turkish citizens that have fled the country in the aftermath of the end of peace talks between the Turkish government and the Kurds, the failed coup d'état, and the prolonged state of emergency. Therefore, the presented study examines the following research questions:*

a) *What reports do asylum-seekers in Germany give about their personal experiences of torture and state terror in Türkiye between 2015-2018?*
b) *What was the mental health burden that arose due to these experiences?*

**Reviewer 1, Comment No. 2**

**The theoretical/conceptual framework is addressed but missing justification and the author seems to use the Introduction/ literature review section as a framework. I think there needs to be a separate and strong section on the theoretical framework which forms a foundation for the study under the figure of framework. Without the theoretical framework, the justification of methodology and method of analysis remain vague and unclear.**

Thank you for this important comment. We have extended the literature review and included a theoretical framework on the United Nations Istanbul Protocol (UNIP), which constituted the basis of our research questions and our interview guide page 4, line 88-108:

*Several studies examined the human rights violations in Türkiye in the aftermath of the coup attempt, focussing on education rights (Aydin & Avincan, 2021), loss of jobs and the freedom to travel (Gul & Gul, 2023), torture and the right to a fair trial (Aydin & Avincan, 2020), the consequences for the Hizmet (Gülen) movement (Pandya et al., 2021), and discrimination and social isolation (Avincan et al., 2023). Aydin and Avincan’s study collected several reports on torture, illegal interrogation techniques, naked body searches, and sexual harassment against all genders, as well as ill-treatment of family members. In a study with Kurdish torture survivors, Dehghan and Osella (2022) examined the psychological impact of sexual torture and revealed the negative consequences for all forms of social relationships and the bodily self. In line with this research, Patel (2019) published an urgent call for more efforts to assess the psychological impact of torture practices on survivors and their families.*

*For a psycho-medical assessment, the Istanbul Protocol on the Effective Investigation and Documentation of Torture and Other Cruel, Inhuman or Degrading Treatment or Punishment provides the most important tool, updated by four civil society organisations and UN anti-torture bodies (1; Iacopino et al., 2022). It offers guidelines for the effective investigation of torture and other inhuman treatment, including general considerations for interviews, the documentation of physical evidence and a psychological evaluation. The Istanbul Protocol consists of the following components: History of ill-treatment, current psychological complaints, post-torture and pre-torture history as well as medical, psychiatric and substance use history, and the assessment of mental status and social function. Over the past 25 years, it has contributed to medical and legal investigations worldwide (Iacopino et al., 2022).*

And page 6, line 141-143:

*The interview questions are directly based on the recommendations of the Istanbul Protocol (1) for the documentation of torture and other ill-treatment.*

**Reviewer 1, Comment No. 3**

**Before moving on to Methods, it would helpful for the readers, if the authors to provide a brief discussion of their own context. This is based on a belief that who you are and how you involved with the topic and study will influence the presentation. We want readers to have a fair understanding of the researcher to make the fullest evaluation of the study and to have greater confidence in what they are about to read. Toward that end, please tell us your relationship to this inquiry. Who are you? What is your interest in this topic? What is your investment in this project? What are your intentions? In addition, your study does not appear to adhere to case study trustworthiness procedures.**

Thank you for this suggestion. We provide more details about our professional background on page 6, line 134-135:

*Our research team consisted of four medical doctors, two of them specialised in forensic medicine, the other two specialised in psychosomatics, and one clinical psychologist.*

**Reviewer 1, Comment No. 4**

**The Discussion is much abbreviated. This section needs a description of your study aims and a brief summary of the findings as they relate to these aims, followed by a comprehensive summary of how your findings compare and contrast to those of previous researchers (including relevant citations) that I only see a few. This will give the paper greater consistency and structural coherence, leaving readers with a clear sense of the paper's aims and trajectory.**

We appreciate the reviewer’s feedback and extend the discussion according to your suggestions:

Page 24, line 617-627:

*This research documents the emotional and physical reactions of survivors of torture and state repression that fled to Germany to seek asylum. Therefore, the presented study aimed at a) assessing personal experiences of torture and state terror in Türkiye between 2015-2018 and b) evaluating the mental health burden that arose due to these experiences.*

*In interviews based on the principles of the Istanbul Protocol, reports of twenty asylum-seekers from Türkiye convey a portrait of a suppressive state that uses illicit means to silence human rights activists and political opponents. Torture and inhuman treatment became a rising concern in detention centres after the failed coup attempt (Aydin & Avincan, Avincan et al., Gul & Gul, 2023).*

**Reviewer 1, Comment No. 5**

**Please provide the limitations for the study**

For a detailed discussion of the limitations, we would like to refer you to the section ‘Limitations’, page 26, line 667-669 and line 676-679. We extended the section as follows:

*‘First of all, the presented experiences of torture and state violence are derived from individual narratives and reflect the personal impressions and insights of the participants that have fled their home country due to life-threatening events. Of course, their reports are shaped by their individual perception of danger and past experiences with discrimination.*

*‘When we conducted the first interviews, we realised that not all interviewees had been imprisoned, as formerly expected, but still every interviewee had experienced aversive and degrading treatment in Türkiye. All of them identified as survivors of torture, defined by them as illicit actions by the state against individuals or groups of people. According to the guidelines provided by the Istanbul Protocol, the severity of the pain and suffering is one of the key elements in defining torture, rather than the nature of the acts inflicted upon the person (30). Therefore, the victim perspective is considered to be crucial in the evaluation of aversive experiences (1).’*

**Reviewer 1, Comment No. 6**

**A few bullet points for implications of the study would be helpful for readers.**

Thank you for this remark. We believe a summary of the implications of the study to be an important asset for any research. We included this in our conclusion, see page 26, line 682-685.

*‘This study is an important contemporary testimony of a relevant period in Turkish history. The collected reports of torture and state repression and its psychological impact send an alarming sign for policy makers to address the prevention of human rights violations and the support needs of survivors from countries where torture and state repression is still practiced today.’*

**Reviewer 1, Comment No. 7**

**I am afraid there are some grammatical and English errors in this paper which make it quite hard to read in places and this is sufficient to make it un-publishable in its present form. The article itself is interesting but it is a difficult read given the issues above. I suggest authors always have their work edited by a professional English editor before submissions. Find someone who is a native speaker of English to proofread your paper.**

Thank you for pointing out the importance of using a clear and precise English language. We have a native speaker proofread the paper.
